# New Tools for Targeted Disruption of Cholinergic Synaptic Transmission in *Drosophila melanogaster*

**DOI:** 10.1371/journal.pone.0064685

**Published:** 2013-05-30

**Authors:** Monica Mejia, Mari D. Heghinian, Frank Marí, Tanja A. Godenschwege

**Affiliations:** 1 Department of Biological Sciences, Florida Atlantic University, Boca Raton, Florida, United States of America; 2 Department of Chemistry & Biochemistry, Florida Atlantic University, Boca Raton, Florida, United States of America; 3 Department of Biological Sciences, Florida Atlantic University, John D Mac Arthur Campus, Jupiter, Florida, United States of America; Columbia University, United States of America

## Abstract

Nicotinic acetylcholine receptors (nAChRs) are pentameric ligand-gated ion channels. The α7 subtype of nAChRs is involved in neurological pathologies such as Parkinson’s disease, Alzheimer’s disease, addiction, epilepsy and autism spectrum disorders. The *Drosophila melanogaster* α7 (Dα7) has the closest sequence homology to the vertebrate α7 subunit and it can form homopentameric receptors just as the vertebrate counterpart. The Dα7 subunits are essential for the function of the Giant Fiber circuit, which mediates the escape response of the fly. To further characterize the receptor function, we generated different missense mutations in the Dα7 nAChR’s ligand binding domain. We characterized the effects of targeted expression of two UAS-constructs carrying a single mutation, D197A and Y195T, as well as a UAS-construct carrying a triple D77T, L117Q, I196P mutation in a Dα7 null mutant and in a wild type background. Expression of the triple mutation was able to restore the function of the circuit in Dα7 null mutants and had no disruptive effects when expressed in wild type. In contrast, both single mutations severely disrupted the synaptic transmission of Dα7-dependent but not glutamatergic or gap junction dependent synapses in wild type background, and did not or only partially rescued the synaptic defects of the null mutant. These observations are consistent with the formation of hybrid receptors, consisting of D197A or Y195T subunits and wild type Dα7 subunits, in which the binding of acetylcholine or acetylcholine-induced conformational changes of the Dα7 receptor are altered and causes inhibition of cholinergic responses. Thus targeted expression of D197A or Y195T can be used to selectively disrupt synaptic transmission of Dα7-dependent synapses in neuronal circuits. Hence, these constructs can be used as tools to study learning and memory or addiction associated behaviors by allowing the manipulation of neuronal processing in the circuits without affecting other cellular signaling.

## Introduction

Nicotinic acetylcholine receptors (nAChRs) are pentameric, membrane-bound ligand-gated ion channels that belong to the Cys-loop superfamily [1], [2], [3], [4], [5]. These receptors are activated by the binding of the neurotransmitter acetylcholine (ACh) or the stimulant alkaloid nicotine, and are permeable to cations such as Na^+^, K^+^ and Ca^2+^. Additionally, these receptors can be modulated by the binding of ligands such a neurotoxins [4], [5]. In vertebrates, there are 17 different subtypes of nAChR subunits, 10 of which are of the α subtype [2], [4], [6], [7]. The α2–10 nAChR subunit subtypes are primarily found in neurons of vertebrate organisms, with homopentameric α7 nAChRs being one of the most prevalent neuronal receptors in the central nervous system [2]. α7 nAChRs are involved in neurological pathologies such as Parkinson’s disease, Alzheimer’s disease, schizophrenia, addiction, epilepsy and autism [2], [3].

Acetylcholine is the primary excitatory neurotransmitter in the central nervous system of insects [1], [8], with glutamate being the primary neurotransmitter at the neuromuscular junctions [9], [10], [11]. There are 10–12 different known insect nAChR subunit subtypes [1], [7]. The insect α7 receptors have been shown to be involved in sensory and cognitive processes [1], memory formation and storage [1], [12], [13], and addiction association behaviors [14], [15], [16], as well as being one of the primary targets of numerous insecticides along with other nAChR subtypes [7]. In *Drosophila melanogaster*, the α7 subunit (Dα7) has a high sequence homology to the vertebrate α7 subunit [17], [18], [19] and it can also form homopentameric receptors [7]. In addition, we have shown that a Dalpha7 dependent synapse can be disrupted with the well-characterized conotoxin ImI as well as MLA, which modulate human alpha 7 receptor function [20], [21].

**Figure 1:**
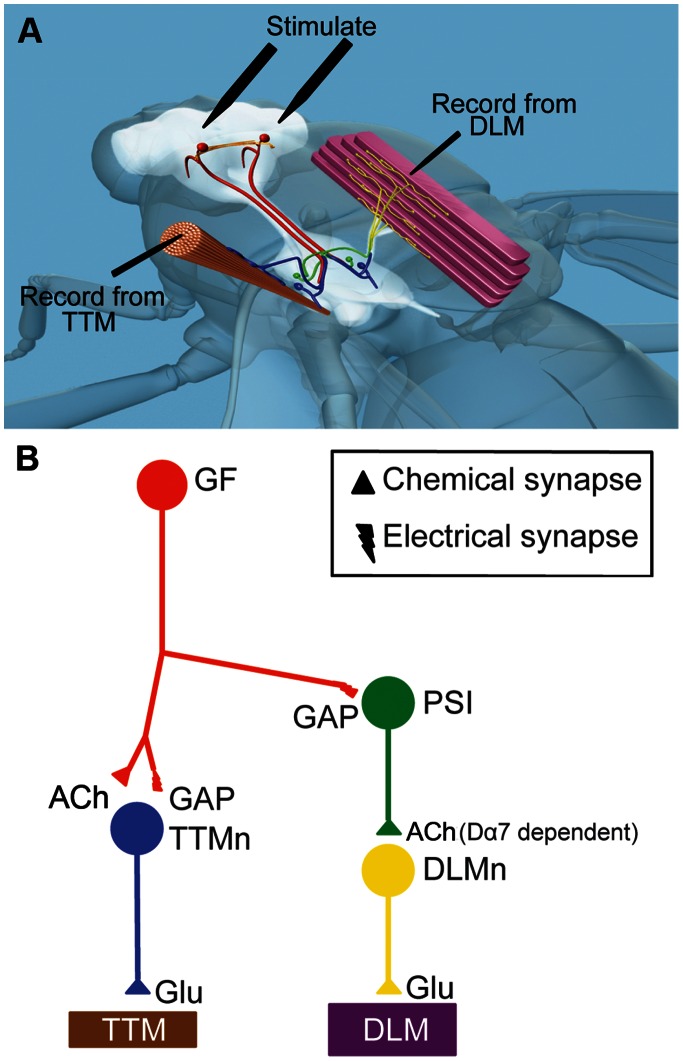
The Giant fiber System (GFS). (A) Depiction of the relative location of the Giant Fiber System (GFS) in the fly. Figure adapted from [20]. Position of the stimulating and recording electrodes for the electrophysiological assays are shown (ground electrode inserted in the abdomen is not shown). (B) Unilateral wriring diagram of GFS neurons. The Giant fiber (red) makes a mixed electrical (GAP) and chemical (cholinergic) synapse onto the Tergo Trochanteral Motorneuron (TTMn, blue), which innervates the jump muscle with glutamatergic synapses (TTMm, brown). The GF also makes an electrical and chemical synapse onto the peripheral synapsing interneuron, which is functionally gap junction dependent (PSI, green, chemical component not shown). The PSI makes a cholinergic connection onto the dorsal longitudinal motorneurons (DLMn, yellow), which innervate the flight muscle with glutamatergic synapses (DLM, purple).

Dα7 is necessary for the function of the Giant Fiber System (GFS, Figure 1) [17], which mediates the escape response of the fruit ﬂy [22]. In the Dα7 null mutants (gfA^PΔEY6^) the GF to Tergo Trochanteral Muscle (TTM) pathway and neuromuscular junctions are unaffected, but no electrophysiological responses can be recorded from the Dorsal Longitudinal Muscle (DLM), when the Giant Fibers (GF) are stimulated indicating a defect in the PSI to DLM connection [17]. The defects in the GF-DLM pathway of Dα7 null mutants were shown to be in the Peripheral Synapsing Interneuron (PSI) to DLM connection and can be fully rescued with expression of Dα7 protein using the UAS-GAL4 system [17], [23].

Here, we manipulated the Dα7 receptor at positions near the ACh binding site in an effort to generate tools for studies related to sensory and cognitive processes, learning and memory, addiction, as well as the testing of Dα7 nAChR modulators.

## Materials and Methods

### Fly Stocks

Fly stocks were kept at either 22**°**C or 25**°**C in vials containing standard media. The following fly lines were used: P[GawB]OK307 (Stock #6488, Bloomington Stock Center; hereafter referred to as A307), gfA^PΔEY6^ (Stock #24879, Bloomington Stock Center). 1–7 day old flies were used in our assays.

### Cloning and Generation of UAS-lines

The Drosophila cDNA of the *Dα7* gene, inserted in the pUAST vector with Bgl-II restriction enzymes, was a generous donation from Dr. Hugo J. Bellen (Baylor College of Medicine, Houston, TX). Point mutations (D77T/L117Q/I196P, Y195T, and D197A) in the Dα7 cDNA were generated by site-directed mutagenesis using the QuickChange Lightning Multi Site-Directed Mutagenesis Kit (KitAgilent Technologies, Inc. Santa Clara CA). The wild type and mutagenized pUAST-Dα7 vectors were sent to The Best Gene Inc. (Chino Hills, CA) for injection into fly embryos to generate UAS-Dα7, UAS-Dα7-D77T/L117Q/I196P, UAS-Dα7-Y195T, and UAS-Dα7-D197A.

### Dα7 Expression in the Giant Fiber System

The UAS-GAL4 system was used to express the Dα7 proteins in the Giant fiber system of the fly [23]. The A307 GAL4 driver line was used to express the wild type and mutant Dα7 UAS-constructs in the Giant Fiber System in the Dα7 null (gfA^PΔEY6^) [17] as well as wild type background.

### Electrophysiology

The methods used to obtain intracellular electrophysiological recordings from the Giant Fiber System have been previously described [20], [21], [24]. For the electrophysiological characterization three parameters were determined for each genotype. Refractory period (RP): twin pulses were used to determine the shortest time between two Giant Fiber (GF) stimuli that result in two Dorsal longitudinal muscle (DLM) responses. Following frequency (FF): the maximum frequency was measured at which the GF to DLM pathway can follow at a one-to-one ratio in response to 10 stimuli. Response latency (RL): an individual stimulus was used to determine the time delay between the stimulation of the GFs and the recording of a response in the TTM or DLM. In addition, the neuromuscular junctions of the GFS were tested by directly activating the DLM and TTM motor neurons via thoracic stimulation as previously described [20], [21], [24].

### Dα7 Receptor Modeling

Molecular models were built of the native Dα7 nAChR and the three mutants using Modeller v9.11 [25] based upon the X-ray crystal structure of the *Aplysia californica* AChBP (PDB ID 2BYP) [26]. Models generated were analyzed using Verify3D [27] and Ramachandran plots were generated in Chimera v1.6.1 [28]. Sequence alignments were generated using Clustal W2 v2.0 [29].

### Statistics

The SigmaPlot software (Systat Software, Inc. San Jose, CA) was used to carry out all statistical analyses. Normality test failed for all groups tested. Thus, a non-parametric Kruskal-Wallis one-way ANOVA with a post-hoc Tukey test was performed for all groups. Additionally, a Mann Whitney Rank Sum test was carried out for comparisons between non-responsive mutant flies (values entered as zero) and wild type flies.

## Results

### Generation of Missense Mutations in Dα7 nAChR

**Figure 2:**
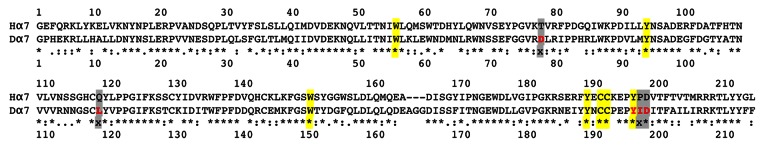
Sequence alignment of ligand-binding domain of Dα7 with Human α7 nAChR subunit. There is a 48% amino acid identity of the entire subunit sequences. Within the ligand-binding domain, there is an 81% amino acid homology, with a 58% amino acid identity. Key residues for agonist binding are highlighted in yellow and mutated residues are in red. An * (asterisk) below residue alignment denotes positions that have a identical residues. A colon denotes groups with strongly similar properties and a scoring of >0.5 in the Gonnet PAM 250 matrix. A period denotes groups with weakly similar properties and a scoring of ≤0.5 in the Gonnet PAM 250 matrix [28].

The whole Drosophila and human α7 subunits (ligand-binding, transmembrane and intracellular domains) have 48% amino acid identity. For the ligand-binding domain, amino acid identity is 58% with 81% homology when similar residues are included in the comparison (Figure 2). In order to manipulate receptor function, we generated three UAS-transgenic lines allowing for the *in vivo* expression of Dα7 carrying the following missense mutations using the UAS-GAL4 system [23]. First, we mutated three non-conserved amino acids in the Dα7 nAChR’s ligand binding domain (LBD) to the human α7 amino acid counterparts (D77T, L117Q, I196P). These amino acids are near the ACh binding site in human α7 receptors [30], [31] and were introduced to test whether these conserved changes would affect Dα7 function. In the second construct, the conserved aspartic acid that is critical for the binding of the well-characterized α-conotoxin TxIA was mutated to an alanine (D197A), a neutral amino acid that should not affect the protein’s folding [32]. Lastly, the conserved tyrosine that is critical for the binding of the well-characterized α-conotoxin ImI, a potent inhibitor of the GF to DLM circuit pathway [20], was mutated to a threonine (Y195T) [30]. These single mutations were designed based upon conotoxin binding studies [33] to produce an inhibitory effect.

### Expression of Wild Type and Mutant Dα7 nAChR Subunits in the GFS of Dα7 Null Mutant Background

When the GFs are stimulated in a wild type fly, the DLMs will respond in a very specific manner. Typically, the GF-DLM pathway’s refractory period determined with twin pulses is ∼5 ms and can reliably follow at one-to-one ratio at ∼100 Hz [24], [34], [35], [36].

**Figure 3:**
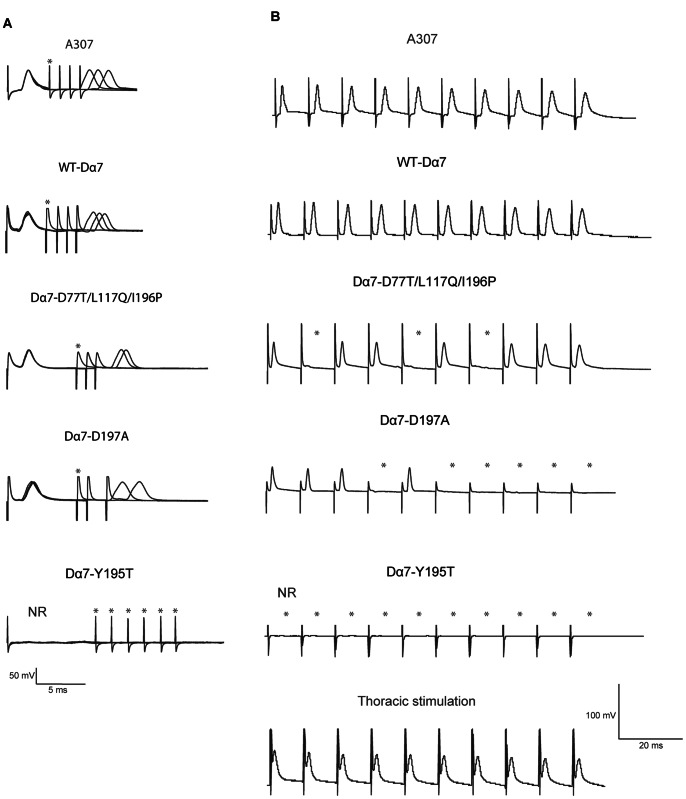
Electrophysiological sample traces of Dα7 nAChR subunits in Dα7 null mutant background. (A) Refractory period (Twin pulses) traces of recordings from the DLM of wildtype control (A307) as well as WT-Dα7, Dα7-D77T/L117Q/I196P, Dα7-D197A and the Dα7-Y195T subunits expressed in a Dα7 null background. (B) Sample traces of recordings from the DLM when the GF was stimulated at 100 Hz stimulation. Genotypes shown are wildtype control (A307) as well as WT-Dα7, Dα7-D77T/L117Q/I196P, Dα7-D197A and the Dα7-Y195T expressed in a Dα7 null background. A trace of direct activation of DLM neurons by thoracic stimulation of a mutant expressing Dα7-Y195T subunits in a Dα7 null background is also shown. n = 24 DLMs for all treatments and genotypes unless otherwise noted. Asterisks indicate lack of responses.

In gfA^PΔEY6^ (Dα7 null) mutants no responses (NR) can be recorded from the DLM when the GFs were stimulated in the brain as previously described [17]. Expression of wild type Dα7 protein in the GFS was able to fully rescue the loss of function phenotype. Here, when the GFs were stimulated with 10 stimuli at 100 Hz the GF to DLM pathway was able to follow reliably at a one-to-one ratio. No significant difference in Refractory Period (RP) and maximum following frequency (FF) was found between the DLM responses of wild type Dα7 rescue flies (RP: 6.04±1.04 ms and maximum FF: 107±23 Hz) and A307 control flies (RP: 4.9±0.7 ms and maximum FF: 110±21.1 Hz, Figure 3).

Similarly, the Dα7 D77T/L117Q/I196P triple mutant restored DLM responses in a gfA^PΔEY6^ background, suggesting that it assembled as functional nAChRs in the Giant Fiber System. However, when the GFs were stimulated with 10 pulses given at 100Hz, the DLMs were only able to follow with 67.6% reliability (Figure 3). We found that the RP (10.04±3.43 ms) was nearly twice as long. The maximum frequency to follow 10 stimuli (FF: 58±16 Hz) was reduced by almost half when compared to the wild type receptor rescue flies (p<0.05, Figure 3).

**Figure 4:**
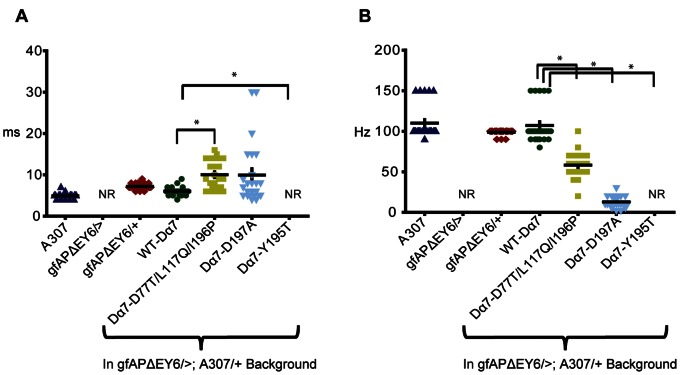
Refractory period and following frequency of Dα7 nAChR subunits in Dα7 null mutant background. (A) Scatterplot of Refractory Periods (Twin pulses) of DLM recordings, when the GF was stimulated in the brain. Genotypes shown are WT-Dα7, Dα7-D77T/L117Q/I196P, Dα7-D197A and the Dα7-Y195T expressed in a Dα7 null background. Wildtype control (A307), negative control gfA^PΔEY6^/>(n = 30 DLMs) and gfA^PΔEY6^/+ flies are shown as well. (B) Scatterplot of maximum Following Frequency of DLM recordings, when the GF was stimulated in the brain. Genotypes are Dα7, Dα7-D77T/L117Q/I196P, Dα7-D197A and the Dα7-Y195T expressed in a Dα7 null background. Wildtype control (A307), negative control gfA^PΔEY6^/>(n = 20 DLMs) and gfA^PΔEY6^/+ flies are shown as well. *p<0.05, n = 24 DLMs for all treatments and genotypes unless otherwise noted.

The D197A mutation also restored responses of the GF to DLM pathway in gfA^PΔEY6^ mutants. However, the reliability of the GF-DLM pathway was severely compromised. The GFs followed only with 17.3% reliability when stimulated at 100 Hz (Figure 3). Here, the GF-DLM RP was 9.96±7.43 ms, which was not statistically significant from the wild type Dα7 rescue flies. However, the maximum FF was only 13±7 Hz (p<0.05 when compared to the wild type Dα7 rescue flies, Figure 4). This suggests that Dα7 subunits with a D197A mutation can assemble a receptor, but it is functionally impaired.

The expression of the Y195T mutant protein did not rescue the gfA^PΔEY6^ Dα7 null mutant electrophysiology phenotype. In these flies, no DLM responses were detected when the GFs of the fly were stimulated via the brain (Figure 3 and 4). By contrast, responses could be recorded when the motorneuron was directly stimulated in the thorax indicating that the glutamatergic neuromuscular junction was not affected by the expression of the mutant subunit (Figure 3). This suggests that Dα7 subunits with an Y195T mutation do not assemble a fully functional ACh receptor at the PSI to DLMn synapse capable of eliciting a postsynaptic potential that is sufficient to trigger an action potential in DLM motor neuron.

### Expression of Wild Type and Mutant Dα7 nAChR Subunits in the GFS of Wild Type Background

**Figure 5:**
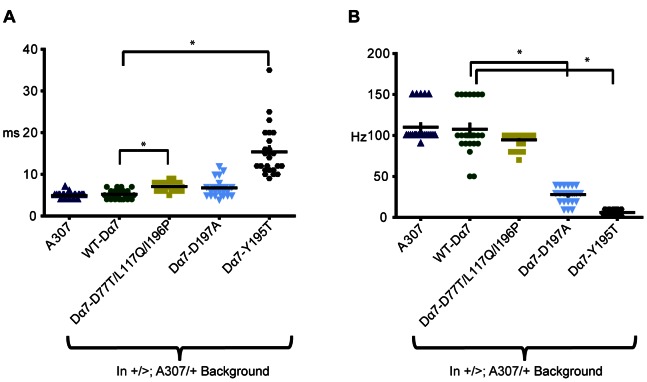
Refractory period and following frequency Dα7 nAChR subunits in wild type background. (A) Scatterplot of Refractory Periods (Twin pulses) of DLM recordings, when the GF was stimulated in the brain. Genotypes shown are WT-Dα7, Dα7-D77T/L117Q/I196P, Dα7-D197A and the Dα7-Y195T expressed in wild type background as well as A307 wildtype control. (B) Scatterplot of Following frequency of DLM recordings, when the GF was stimulated in the brain. Genotypes shown are WT-Dα7, Dα7-D77T/L117Q/I196P, Dα7-D197A and the Dα7-Y195T expressed in wild type background as well as A307 wildtype control. *p<0.05, n = 24 DLMs for all treatments and genotypes.

We expressed wild type and mutant Dα7 proteins in wild type background in order to assess any disruptive effects on the function of the neuronal circuit. Overexpression of the wild type Dα7 protein in a wild type background had no effect on the circuit function when compared to the A307 wild type controls. The GF to DLM pathway followed reliably at a one-to-one ratio when the GFs were stimulated at 100 Hz. Here, the maximum FF was 108±31 Hz, and the RP remained unaffected (5.21±1.10 ms, Figure 5). Although the D77T/L117Q/I196P triple mutant protein slightly increased the GF-DLM refractory period when compared to wild type Dα7 overexpression (7.08±1.06 ms p<0.05), it had no statistically significant effects on the reliability of the GF-DLM pathway (FF: 95±9 Hz, Figure 5).

Conversely, the expression of the D197A mutant protein in a wild type background had no effect on the GF-DLM RP (6.75±2.09 ms) but it strongly reduced the reliability of the GF-DLM pathways when stimulated at high frequencies (FF: 28±10 Hz, p<0.05, Figure 5). This suggests that the Dα7 D197A subunits co-assembled with the wild type subunits, but the function of the hybrid nAChRs was impaired.

Finally, expression of the Y195T mutant in a wild type background disrupted the GF to DLM pathway in every tested specimen. The RP that was nearly three times longer than wild type controls (15.38±6.08 ms, p<0.05) and the ability to follow stimuli at high frequency was almost abolished (FF: 6±3 Hz, p<0.05, Figure 5). This suggests that Dα7-Y195T is well expressed and can effectively disrupt the receptor function by the assembly of Dα7-Y195T/Dα7-WT hybrid receptor in wild type background flies.

Finally, it should be noted that different transgene insertions for every mutant genotype were generated. Five insertions from each transgenic line were tested for the presence and reliability of the DLM response, and no major differences between the different transgenes were detected (data not shown). This implies that the phenotypes described here were not due to differential dosage expression of the proteins by the transgenes. Therefore, the inability of expression of Dα7-Y195T subunits in Dα7 null mutants to restore DLM responses is not due to lack of expression, dosage, protein transport or assembly of the mutant protein.

### Effects of Dα7 Mutant Proteins on Gap Junctions and Glutamatergic Synapses

The monosynaptic GF to Tergo trochanteral motor neuron (TTMn) connection is a mixed electrical (GAP junction) and chemical (ACh) synapse. The GF-TTM response latency in wild type flies consistently falls between 0.8 to 1.0 ms [24], [34], [35], [36], [37]. At the GF-TTMn connection the electrical synapse is dominant, because the GF-TTM response latency increases from ∼0.8 ms to ∼1.2–1.8 ms [37], [38], [39] in gap junction mutants but not when the chemical synapse is disrupted [17], [40], [41]. When we compared the response latencies of the GF-TTM pathway of the expression of WT-Dα7 (0.95+/−0.09 ms) with the expression of the D197A (0.88+/−0.1 ms), Y195T (0.97+/−0.15 ms) and D77T/L117Q/I196P (0.93+/−0.12 ms) mutant subunits in wild type background we did not find a significant difference. This suggests that the expression of the mutant Dα7 subunits has no disruptive effect on the function of electrical synapses.

Additionally, when we tested the glutamatergic neuromuscular junctions (NMJs) of the expression of mutant subunits in wildtype and gfA^PΔEY6^ background there was no noticeable difference in the DLM amplitudes seen when compared to expression of WT-Dα7 (Figure 2 and data not shown). In addition, in any tested case of absent DLM responses when the GF was stimulated, responses could be obtained when the DLMns where activated directly by thoracic stimulation (Figure 2). This suggests that the expression of the mutant Dα7 subunits does not interfere with the function of glutamate receptors.

## Discussion

The Dα7 subunit has been shown to be essential for the function of PSI-DLMn connection in the Giant Fiber System [17]. Here we investigated the role of D197A, Y195T and D77T/L117Q/I196P missense mutations in the ligand-binding domain of Dα7 nAChRs on the function of Giant Fiber circuit. All three mutant subunits were either able to restore the circuit function in the Dα7 null background or efficiently disrupt the function of wild type nAChRs in wild type background demonstrating that all three mutant subunits are expressed, transported and assembled in vivo. The finding that the expression D197A and Y195T but not D77T/L117Q/I196P subunits had strong poisonous effects on the circuit function in wild type background suggests that they assemble receptors with wildtype Dα7 subunits. However, these hybrid receptors are not working properly in response to endogenous ACh release when the circuit is stimulated. In Drosophila, the Dα7 subunit can form homopentameric as well as heteropentameric receptors with Dα5 subunits [7]. However, it is unknown whether the Dα5 or any other subunits are expressed at the PSI-DLMn synapse.

**Figure 6:**
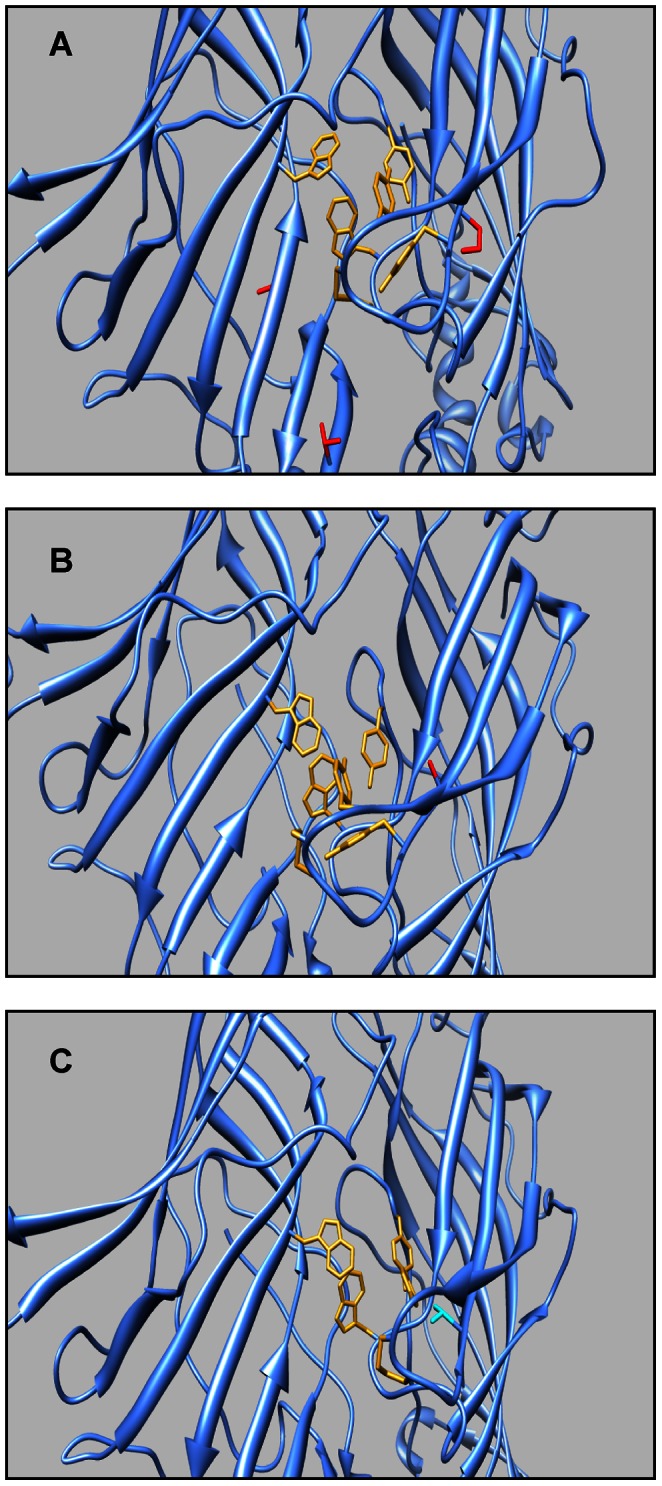
Structural models of mutant Dα7 nAChR ligand-binding domain. (A) Triple mutant D77T/L117Q/I196P: key agonist binding residues (yellow) and point mutations (red) are highlighted. (B) D197A mutant: key agonist binding residues (yellow) and single point mutation (red) are highlighted. (C) Y195T mutant: single point mutation (light blue) overlapping one key agonist binding residue and other key agonist binding residues (yellow) are highlighted.

The location of mutated residues in the Dα7 receptor can be visualized in models of ligand-binding domains (Figure 6) that we built based upon the acetylcholine binding protein (AChBP). AChBPs are well-characterized homologs of the extracellular domain of nAChRs and a useful tool for evaluating the structural characteristics of agonist/inhibitor binding [42]. X-ray crystal structures of AChBP/human α7 chimera bound with an agonist showed that ligand recognition and binding is accomplished by a series of highly conserved residues between the human α7, AChBP, and Dα7. The primary residues for agonist binding have been identified to be Trp 56, Tyr 94, Trp 151, Tyr 190, Cys 192, Cys 193, and Tyr 197 (Figure 2, yellow highlights) [43]. It can be hypothesized that any dramatic changes in amino acid structure within this ligand recognition/binding site may cause an interruption of agonist binding, resulting in a non-functional receptor. The triple mutations along with the primary binding residues are highlighted in Figure 6a. These three mutations (D77T/L117Q/I196P) were introduced due to their presence near the ligand binding domain and their complete lack of homology to the human α7 amino acid counterpart. From the model, it can be seen that the mutations (red) had very little effect on the structure of ligand recognition/binding residues. This reinforces the ability of this mutant subunit to restore GF-DLM pathway, as an agonist can still bind to the receptor and induce the conformational changes necessary for proper receptor function. The electrophysiology data shows that the Dα7 D77T/L117Q/I196P triple mutant rescues the mutant phenotype to an extent in a Dα7 null background. Although the mutant subunits had a slight effect on the refractory period of the GF-DLM when expressed in a wild type background, they did not have a deleterious effect on the reliability (following frequency) of the GF-DLM pathway. This confirms the formation of fully functional receptors and suggests the mutant subunits can be either homopentameric or co-assemble with the wild type subunits.

The Dα7 D197A mutant, shown in figure 6b, highlights the change from Asp to Ala, a negatively charged to an uncharged residue. This single point mutation can potentially have tremendous effects on normal receptor function. As seen in the molecular model, Asp 197 is not a primary ligand recognition residue; however, it is part of the C-loop, which has been shown to be primarily involved in ligand binding. It has been suggested that the residues in this C-loop that are not directly involved in ligand binding still contribute to receptor affinity towards an agonist via transduction of binding to channel gating [43]. Therefore, the mutation of a charged residue to an uncharged residue in this integral C-loop region can have a profound effect on Dα7 function and this is observed when the mutants were tested electrophysiologically. A DLM response was detected, however both the refractory period and the reliability of the GF-DLM were severely compromised when these mutants were expressed in a Dα7 null background. Based on this data, it is clear that the protein subunits are being expressed, but only partially rescuing the pathway function. When the Dα7 D197A mutant protein was expressed in a wild type Dα7 background, they had no significant effect on the GF-DLM refractory period, but the reliability of the pathway (following frequency) was harshly distressed. This data suggests that the improper electrophysiology rescue is not due to expression dosage, transportation or assembly issues and supports the conclusion that this point mutation may be disrupting the receptors ability to transduce ligand binding to channel opening and closing. As with the triple mutant, these mutant subunits may form homopentameric receptors or co-assemble with the wild type subunits.The Dα7 Y195T mutants introduce a single point mutation to a key amino acid residue for agonist binding (Figure 6c). It is part of the integral C-loop and it has been shown to have direct van der Waals interactions with the agonist and other key amino acids within the ligand-binding domain [43]. Therefore, it can be hypothesized that the mutation of this Tyr residue to a Thr disrupts the conserved binding pocket of the receptor, causing a change in the affinity for the agonist and a change in proper receptor function. When these mutants were expressed in a Dα7 null background no DLM responses were detected. This lack of rescue is not due to the protein not being expressed or at such low concentrations that they are unable to drive the synapse, given that when the mutant protein was expressed in a wild type background it had a great poisonous effect on the functionality of the GFS. This suggests not only that the proteins are being expressed at an appropriate dosage, but also that they can be transported and assembled correctly into nAChRs. Furthermore, the fact that Y195T does not restore any responses in the Dα7 null mutant background, but acts as a poisonous subunit in the wild type background, suggests that, unlike the other two mutations created, there is no co-assembly with other subunits except the wild type Dα7.

Finally, several tactics have been employed to distress the neuronal circuitries in the fly in order to study the mechanisms of learning and memory. Examples of these strategies are the overexpression of endogenous proteins such as TAU [44] and notch [45], the ablation of the mushroom bodies [46], [47] and the use of the temperature sensitive dynamin mutant gene *shibire^ts^* paired with the UAS-GAL4 system [48], [49]. However, most of these tools have developmental effects or affect other cellular processes along with circuit function. Expression of the mutant Dα7 subunits seem to specifically affect only cholinergic transmission without affecting other signaling mechanisms. Therefore, the transgenic lines described here, which allow targeted expression, may provide a novel in vivo tool to manipulate circuit function and their associated behaviors to different degrees. In addition, nAChR structure/function relationships are not well characterized in vivo in many model systems. These mutants provide an example of functionally induced changes that can be used to characterize in vivo the role of AChRs, which can lead to further understanding of cholinergic neurological processes.
